# Association of biosecurity and hygiene practices with avian influenza A/H5 and A/H9 virus infections in turkey farms

**DOI:** 10.3389/fvets.2024.1319618

**Published:** 2024-03-14

**Authors:** Ariful Islam, Monjurul Islam, Pronesh Dutta, Md Ashiqur Rahman, Abdullah Al Mamun, AKM Dawlat Khan, Mohammed Abdus Samad, Mohammad Mahmudul Hassan, Mohammed Ziaur Rahman, Tahmina Shirin

**Affiliations:** ^1^EcoHealth Alliance, New York, NY, United States; ^2^School of Life and Environmental Sciences, Deakin University, Geelong, VIC, Australia; ^3^Institute of Epidemiology, Disease Control and Research (IEDCR), Dhaka, Bangladesh; ^4^National Reference Laboratory for Avian Influenza, Bangladesh Livestock Research Institute (BLRI), Savar, Bangladesh; ^5^Queensland Alliance for One Health Sciences, School of Veterinary Science, University of Queensland, Gatton, QLD, Australia; ^6^Faculty of Veterinary Medicine, Chattogram Veterinary and Animal Sciences University, Chattogram, Bangladesh; ^7^One Health Laboratory, International Centre for Diarrheal Diseases Research, Bangladesh (ICDDR,B), Dhaka, Bangladesh

**Keywords:** prevalence, risk factors, HPAI H5N1, H9N2, poultry, zoonotic

## Abstract

High pathogenicity avian influenza (HPAI) H5N1 outbreaks pose a significant threat to the health of livestock, wildlife, and humans. Avian influenza viruses (AIVs) are enzootic in poultry in many countries, including Bangladesh, necessitating improved farm biosecurity measures. However, the comprehension of biosecurity and hygiene practices, as well as the infection of AIV in turkey farms, are poorly understood in Bangladesh. Therefore, we conducted this study to determine the prevalence of AIV subtypes and their association with biosecurity and hygiene practices in turkey farms. We collected oropharyngeal and cloacal swabs from individual turkeys from 197 farms across 9 districts in Bangladesh from March to August 2019. We tested the swab samples for the AIV matrix gene (M gene) followed by H5, H7, and H9 subtypes using real-time reverse transcriptase-polymerase chain reaction (rRT-PCR). We found 24.68% (95% CI:21.54–28.04) of turkey samples were AIV positive, followed by 5.95% (95% CI: 4.33–7.97) for H5, 6.81% (95% CI: 5.06–8.93) for H9 subtype and no A/H7 was found. Using a generalized linear mixed model, we determined 10 significant risk factors associated with AIV circulation in turkey farms. We found that the absence of sick turkeys, the presence of footbaths, the absence of nearby poultry farms, concrete flooring, and the avoidance of mixing newly purchased turkeys with existing stock can substantially reduce the risk of AIV circulation in turkey farms (odds ratio ranging from 0.02 to 0.08). Furthermore, the absence of nearby live bird markets, limiting wild bird access, no visitor access, improved floor cleaning frequency, and equipment disinfection practices also had a substantial impact on lowering the AIV risk in the farms (odds ratio ranging from 0.10 to 0.13). The results of our study underscore the importance of implementing feasible and cost-effective biosecurity measures aimed at reducing AIV transmission in turkey farms. Particularly in resource-constrained environments such as Bangladesh, such findings might assist governmental entities in enhancing biosecurity protocols within their poultry sector, hence mitigating and potentially averting the transmission of AIV and spillover to humans.

## 1 Introduction

The global attention on high pathogenicity avian influenza (HPAI) H5Nx has intensified due to its significant impact on various aspects such as poultry production, trade, food security, human health, and economic losses (1, 2). The recent devastating outbreak of HPAI H5Nx in diverse poultry species, including chicken, duck, turkeys, gallinaceous birds, and wild birds, with occasional spillover into mammals, including humans, has further underscored the urgency of addressing this issue (3, 4). There have been increased reports of both HPAI and low pathogenicity avian influenza (LPAI) in the Southeast Asian poultry industry (5, 6), including Bangladesh, affecting various poultry production systems (7, 8). Since the first reported HPAI H5N1 outbreak in poultry in Bangladesh (9), more than 580 HPAI H5N1 outbreaks have been recorded in poultry and wild birds in Bangladesh (10, 11), where the maximum cases originated from commercial poultry farms (12). In Bangladesh, people come into close contact with poultry in different production systems (13, 14), which enhances the transmission of avian influenza viruses (AIVs) from birds to humans. In addition to poultry, 8 H5N1 and 3 H9N2 cases have been reported in humans, including one fatal case, where they have reported direct contact with sick poultry (15, 16).

Bangladesh is one of the world’s most densely populated nations, with 160 million people as residents and a population density of 1,072 individuals per square kilometer (17). It is also considered a low- to middle-income country in South Asia, with an agriculture and livestock-based economy (18). The total number of poultry in Bangladesh is anticipated to be 258.22 million, with 189.26 million chickens, 67.52 million ducks, and 1.44 million turkeys (19). The domestication of turkeys has considerably enhanced the worldwide population’s nutritional and financial status (20). Turkeys have delicious and high-quality meat, for which they are commonly reared worldwide. Although it was previously considered a festive food, especially at Christmas, its demand has increased over the years because of its popularity as a protein source (21). Turkey meat is the second most popular poultry meat consumed after chicken, reporting 6.3 million tons in 2016 globally, reflecting an increasing trend in the poultry market (22). Turkey meat has a comparatively low percentage of fat and a high rate of proteins compared to other types of meat, resulting in a higher demand for turkeys as the global demand for white meat increases (23, 24). Turkey rearing in Bangladesh started in 2002 by importing some varieties of birds brought from India (25). This farming gained popularity after 2016, and interested farmers started turkey farming by importing day-old turkey chicks from India (26). Turkey grows faster than broiler chickens and becomes suitable for slaughter purposes within a very short time, making meat production the primary focus over egg production in Bangladesh (27). As turkey farming gained popularity, many new farmers entered the industry, lacking essential knowledge in feeding, housing, disease prevention and management, standard growth patterns, feed efficiency, and hatching egg incubation (26). However, this sector experienced a sudden decline in 2019, with one of the probable causes being the spread of multiple infectious diseases, including AIV (28). In addition, turkeys are more vulnerable to AIV infection than other poultry species (29, 30). Previous studies have detected the circulation of both H5N1 and H9N2 virus subtypes in turkeys, observed in farms and live bird markets (LBMs) in Bangladesh (31–33).

In Bangladesh, turkeys are reared in a free-range and semi-intensive system (26), facilitating potential contact with other backyard poultry species and wild birds, which can enhance the chance of AIV transmission (34, 35). Additionally, farmers often lack knowledge about turkey rearing, biosecurity, and disease prevention, which could heighten the risk (27, 36). LBMs also serve as potential hotspots for AIV transmission, providing an environment for the maintenance of the virus throughout the year and increasing the likelihood of new strains emerging (37, 38). These markets are commonplace for selling different bird species sourced from both commercial and backyard poultry farms (39). The co-circulation of HPAI and LPAI strains in different poultry-rearing systems in Bangladesh (7, 32, 40) poses significant challenges to successful turkey farming. In 2017, the abrupt death of turkeys was reported on a turkey farm in the Mymensingh district of Bangladesh, where several birds of that farm were identified as positive with HPAI H5N1 of clade 2.3.2.1a with a 13% mortality rate (28, 36).

The circulation of AIV subtypes and biosecurity practices in turkey farms are poorly understood in Bangladesh. However, a lack of knowledge regarding turkey rearing, housing, and disease prevention practices among poultry raisers influences the reluctance to implement biosecurity practices in these farms. Therefore, the study aimed to identify H5, H7, and H9 subtypes of AIV in turkey farms and assess the influence of biosecurity and hygienic practices on AIV transmission. The study also aimed to provide recommendations for improving biosecurity practices to reduce AIV and its subtype infections in farms and prevent spillover to humans.

## 2 Methodology

### 2.1 Ethical approval

The research was authorized by both the Animal Experimentation Ethics Committee and the Ethics Committee at the Chattogram Veterinary and Animal Sciences University (Protocol: CVASU/Dir(R&E) EC/2019/126(1)).

### 2.2 Study design, data, and sample collection

**Figure 1:**
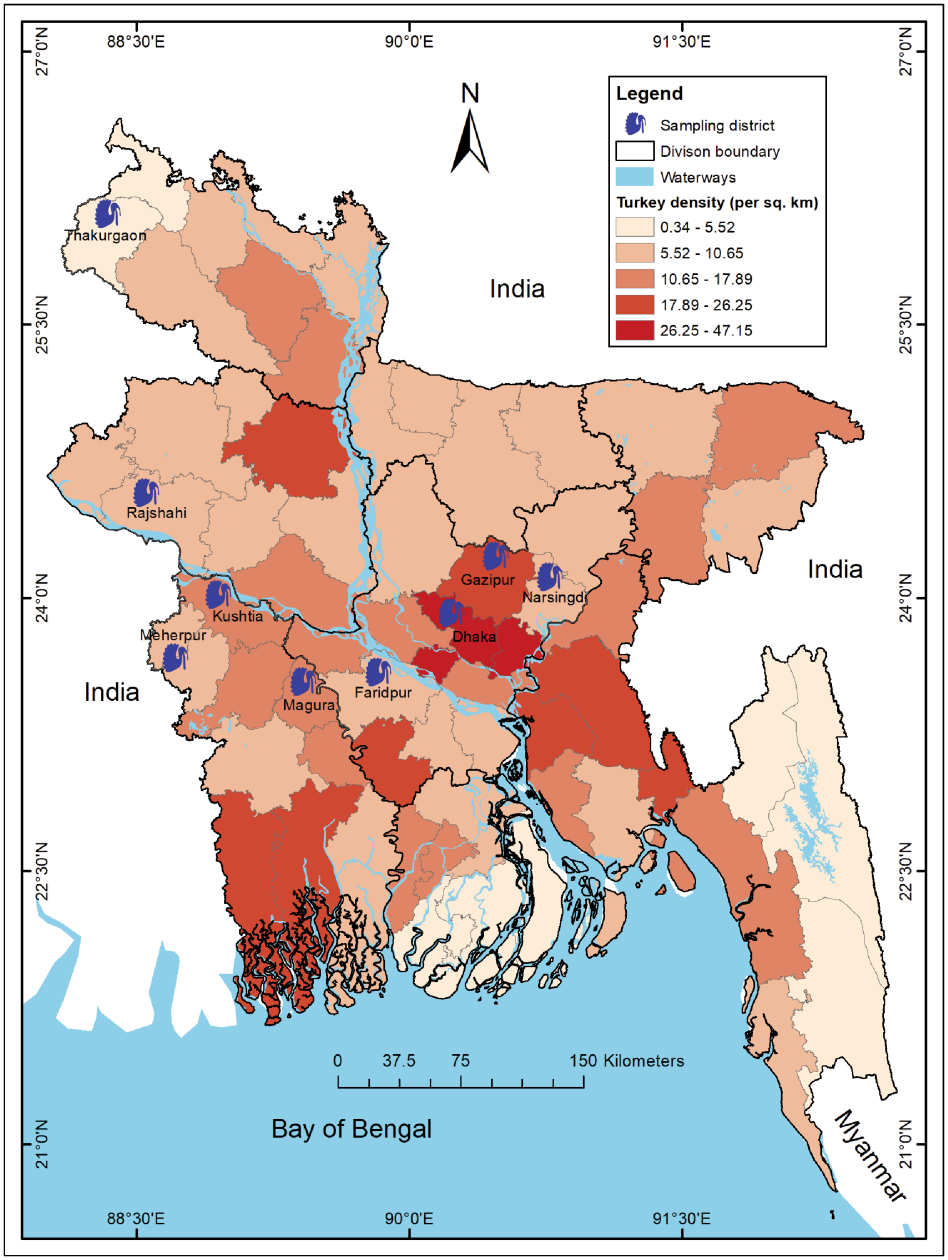
Map of Bangladesh showing study districts of turkey farms during 2019. The intensity of color gradient represents the distribution of turkey density (per sq. km) in 64 districts across Bangladesh and 9 blue turkey symbols indicate the sampling districts. The map was generated using ArcGIS v10.8 (ESRI, Redlands, CA, United States) with freely available shape files obtained from DIVA-GIS. (https://www.diva-gis.org/).

We conducted a cross-sectional study in 197 turkey farms across 9 districts (out of 64) of Bangladesh from March to August 2019. We selected the farms and study areas based on turkey density and trading regions (19, 33) (Figure 1).

We sampled an average of 4 turkeys per farm, with a range of 2 to 6 turkeys, depending on the size of the farm and consent of the farmer. The number of turkeys sampled per farm varied across study areas due to differences in farm size and the willingness of the farmers to provide turkeys for sampling. The selection of turkey was random, and no specific characteristics were considered during sampling. We recorded the health status of turkey birds following the selection process and identified the sick birds based on their clinical signs and symptoms (36, 41).

We collected pooled oropharyngeal and cloacal swabs from each turkey in a 1.8-mL sterile cryovial containing 1 mL of viral transport media (VTM) composed of Hank’s balanced salt solution (ICN Biomedicals, Inc., United States), 2% bovine albumin, with a pH of 7.4 containing amphotericin B (15 μg/mL), penicillin G (100 units/mL), and streptomycin (50 μg/mL) (42, 43). During sampling within farms, we kept the swabs in VTM in a cool box at approximately 4°C. The samples were then transferred to a portable dry shipper with a temperature setting at −196°C. We stored the samples in a −80°C freezer in the laboratory until testing.

**Figure 2:**
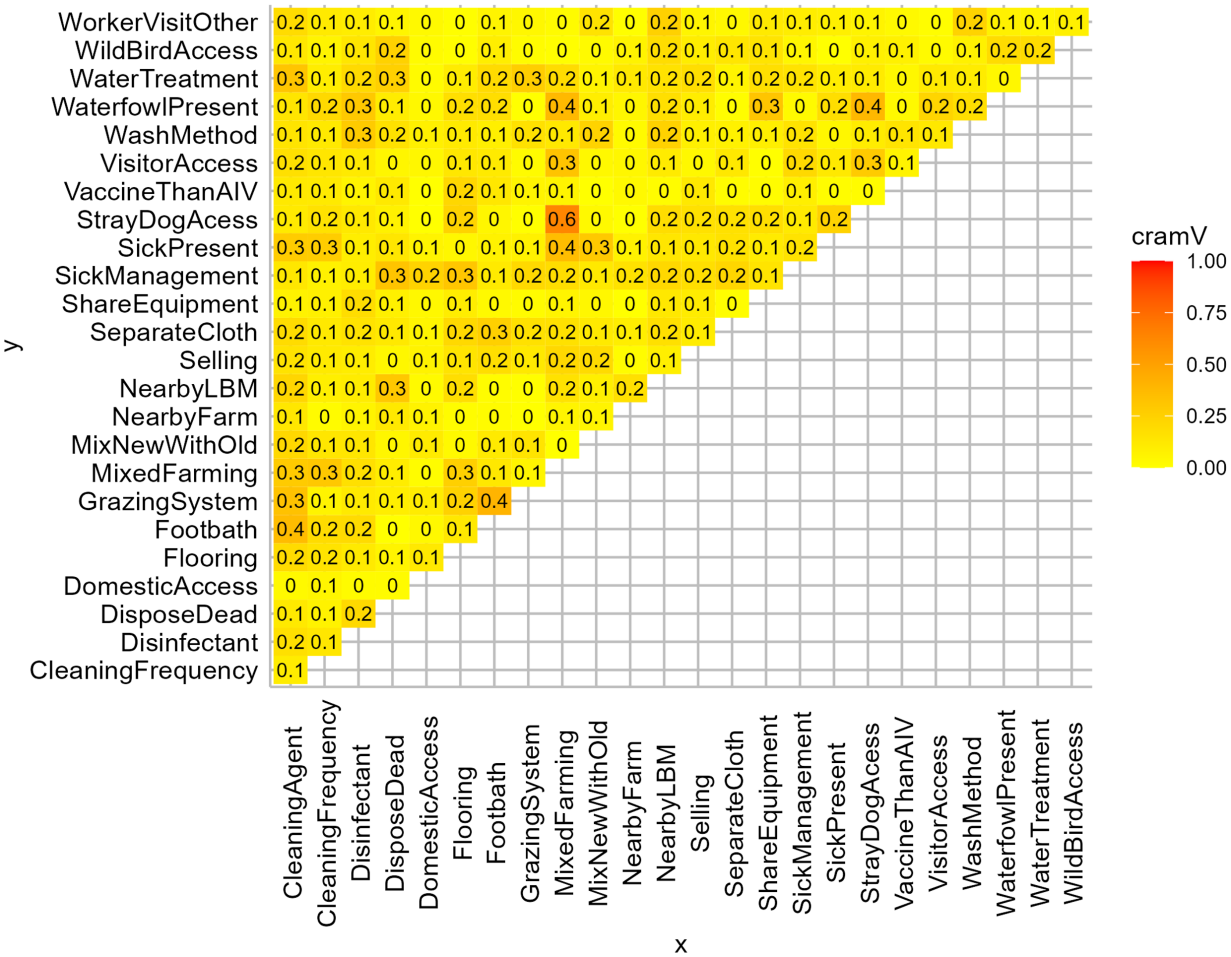
Matrix plot of Cramer’s V of all independent variables. Based on Cramer’s V > 0.3, MixedFarming, GrazingSystem, CleaningAgent, WaterfowlPresent, DisposeDead were removed from the analysis.

**Figure 3:**
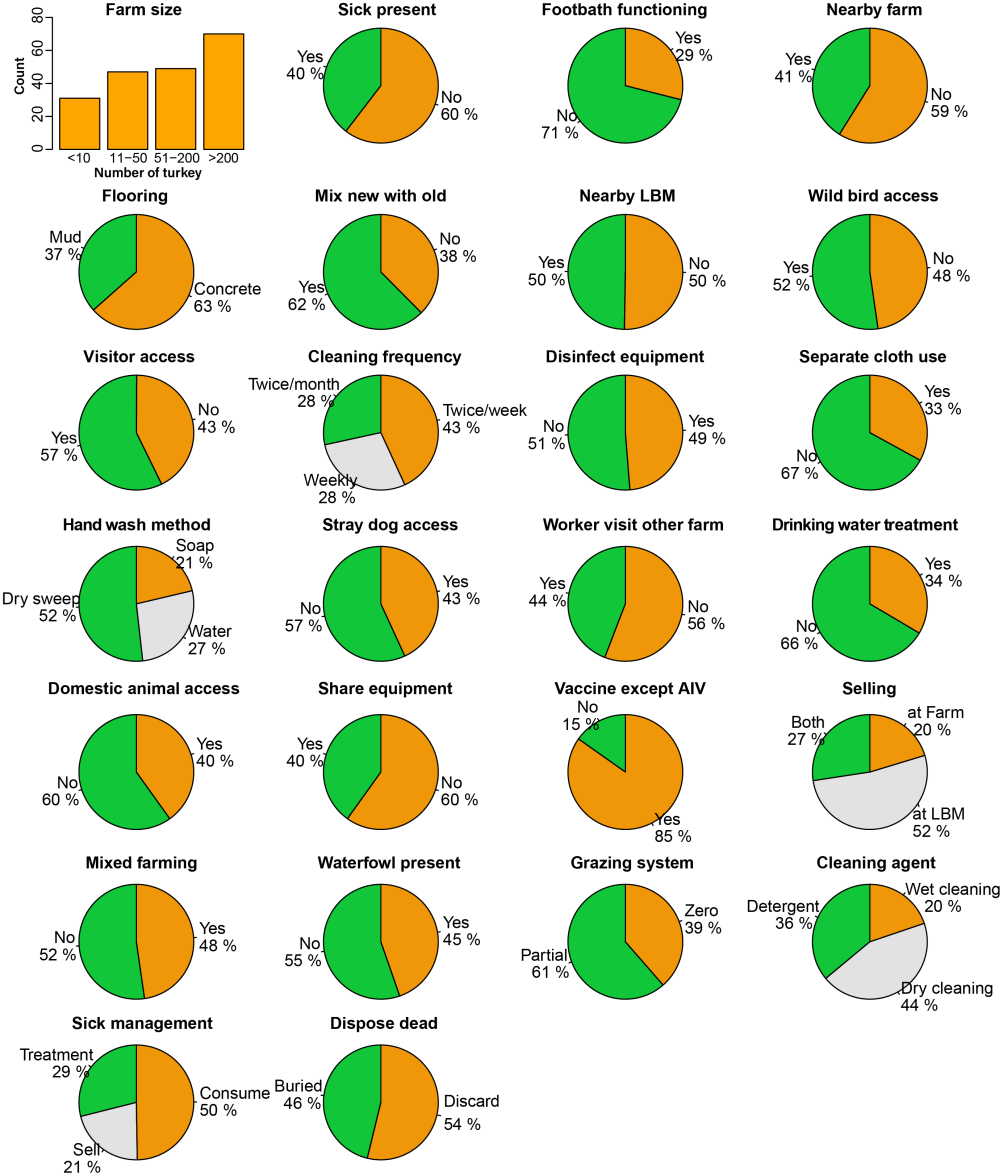
Frequency distribution of biosecurity and hygienic practices and physiographic characteristics of turkey farms.

We prepared a structured questionnaire to collect information about farmers’ demography, farm characteristics, farm management, hygiene, and biosecurity practices. The questionnaire followed the guidelines of the Department of Livestock Service (DLS), Bangladesh (44). We administered the questionnaire, which included 25 variables that might be associated with AIV and constitute a potential risk factor (Figures 2, 3).

### 2.3 Laboratory testing

The pooled oropharyngeal and cloacal swabs from each bird were analyzed to detect the AIV Matrix (M) gene. The magnetic bead-based RNA isolation approach was used to extract RNA using the MagMAXTM-96 AI/ND viral RNA isolation kit (Applied Biosystems™, San Francisco, CA) in a KingFisher™ Flex 96-well robot (Thermo Scientific™, Waltham, MA) in accordance with the instructions provided by the manufacturer. Initially, we examined the swab samples for M-gene using real-time reverse transcriptase PCR (rRT-PCR) assay with reference primers and probes, as described by Spackman et al. (45). The samples that yielded a positive result for the M gene were then subtyped for the H5, H7, and H9 strains with hemagglutinin gene-specific primers and probes using rRT-PCR assay (46, 47). If the cycle threshold (Ct) < 40, we determined that the samples tested AIV M-gene positive, and if Ct < 38, then H5 positive (48). We classified positive samples as A HA/Untyped if they tested positive for the M gene but negative for the H5, H7, and H9 genes.

### 2.4 Statistical analyses

We investigated the effect of farm-level biosecurity practices against AIV presence in farms using generalized linear models in R version 4.2.0 within RStudio version 2022.02.2 (49). The binomial exact test was used to find the 95% confidence interval (CI) along with the prevalence of AIV (M-gene) and its subtypes of H5 and H9. We deployed the pie function to illustrate the frequency distributions of the farm biosecurity variables through pie charts. We calculated Cramer’s V coefficients among all independent variables to identify potential multicollinearity (Figure 2). Based on the threshold of Cramer’s V > 0.3 (50), we excluded the variables: mixed farming, grazing system, cleaning agent, waterfowl present, and disposal of dead. Then, we fitted a Generalized Linear Mixed-effect logistic model (GLMM) using the R package lme4 to evaluate the impact of biosecurity practices on AIV transmission in farms. The response variable was “AIV infection presence/absence at the farms” (AIV infected indicating at least one positive bird in the farm), while the model included 20 explanatory variables to identify significant risk factors among them. To capture all the unobserved subject-specific characteristics, we incorporated sampling events (sampling region of farms) and number of samples per farm as random effects for the model. The application sjPlot package was used to generate plots of the Odds ratios for the model estimates. After the multivariable model, we conducted post-hoc pairwise comparisons using the emmeans package (51) to get the marginal means of significant explanatory variables.

## 3 Results

### 3.1 Socio-demographic characteristics of turkey farm owners

**Figure 4:**
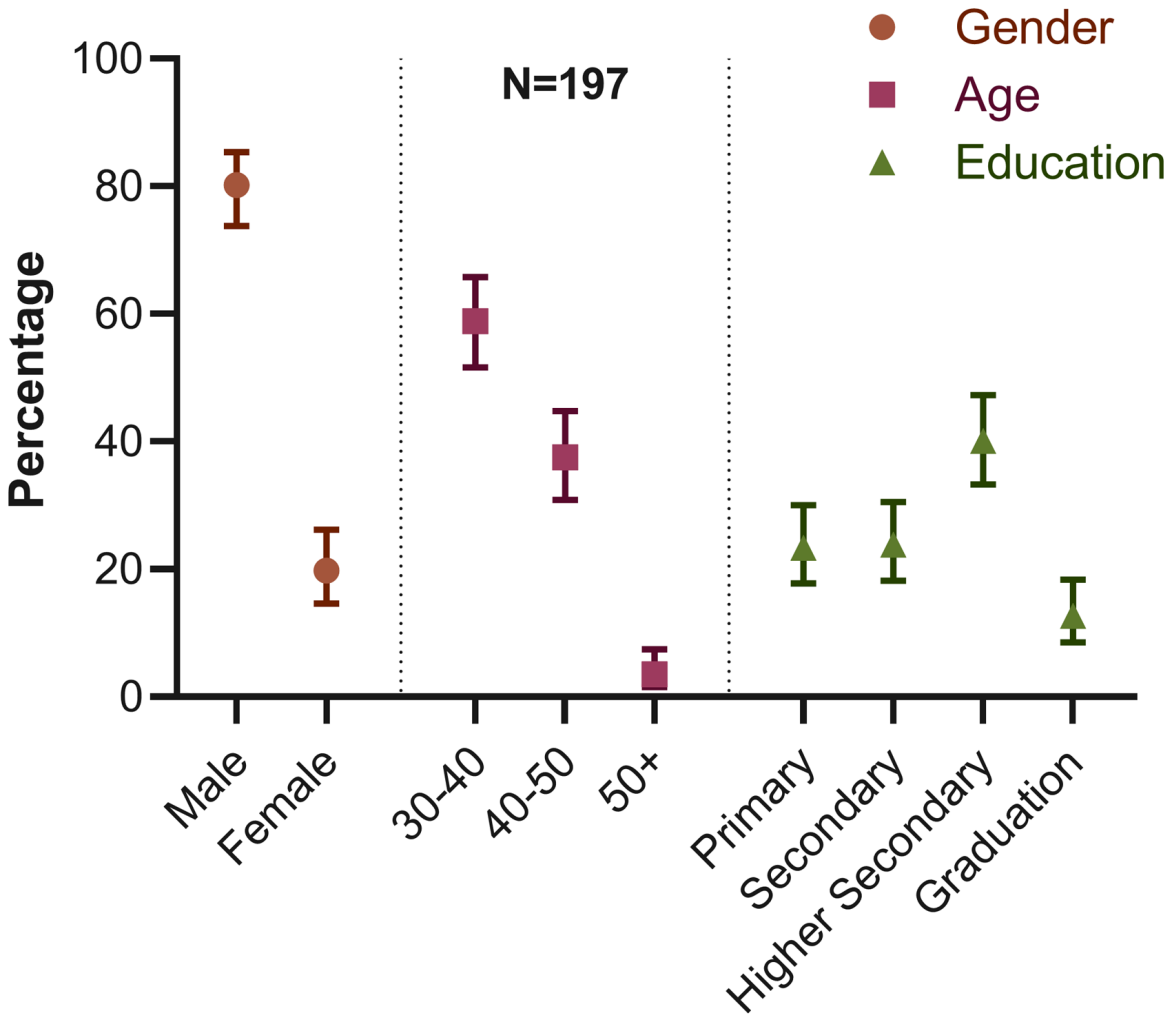
Distribution of demographic characteristics of farm owners (percentage and 95% confidence interval for each variable).

We recorded the demographic characteristics of all 197 turkey farms. In our study, we found that most farm owners were men (80.2%; 95% CI: 73.81–85.39) (Figure 4). Most farm owners were between the ages of 30 and 40 (58.88%; 95% CI: 51.65–65.76). Only a small portion, 3.55% (95% CI: 1.57–7.48) of the owners were 50 years or older. We also discovered that 40.1% of the owners had finished their higher secondary education (12th grade). The percentage of owners with primary education or less was comparable to those with secondary education (10th grade), ranging between 23 and 24%. In contrast, only 12.69% (95% CI: 8.53–18.35) of the owners had graduated.

### 3.2 Biosecurity and hygienic practices and physiographic characteristics of the turkey farms

We found that the variables pertaining to biosecurity, hygiene practices, and farm characteristics showed a nearly uniform distribution across all investigated 197 farms. A considerable number of farms implemented notable good practices, including 85% of the farms vaccinating their turkeys against infectious diseases such as Newcastle disease and Infectious bursal disease, excluding AIV as part of their disease prevention measures (Figure 3).

The floor cleaning frequency was also fair enough, with 43% of the farms cleaning the floor at least twice a week, 28% of farms cleaning weekly, and the remaining 28% cleaning twice a month. We also noticed inadequate biosecurity measures in certain categories. For instance, 71% of the farms had no footbath at the entry, which increases the risk of transmitting infectious diseases to the farms. In addition, we found that 50% of farmers slaughtered and consumed sick poultry, while 21% sold the sick poultry and only 29% treated the poultry as per standard practice. We observed that 44% of farmers performed dry cleaning, using only a broom without any cleaning agent, while 20% relied solely on water and 36% used detergent as a disinfectant during floor cleaning. Some factors involved the risk of virus spillover from poultry to humans, such as hand washing method after handling poultry; only 21% of farmers washed their hands using soap, while 27% used water, and the majority (52%) just wiped their hands with cloths or towels. We found diverse selling patterns of live poultry and eggs from the farm, with 52% selling their products at LBMs, 20% at other farms, and 27% at both locations.

We observed several characteristics that occurred with similar frequency among the farms, for example, rearing other species with turkey (48%), the presence of nearby LBM (50%) and farms (41%), waterfowl presence or access in the farm premises (45%), wild bird access to the farm (52%), visitor access to the farm (57%), disinfectant used for cleaning equipment (49%), stray dog access to the farm (43%), and domestic animal access to the farm (40%). We also monitored some practices that could transmit viruses from other poultry, as 40% shared their equipment with other farms or LBMs, and 62% used to mix newly purchased poultry with the old ones. On the day we sampled, 40% of farms had sick poultry present. Alarmingly, we found that 54% of farms openly discarded their dead poultry on the farm premises, which is considered poor biosecurity practice.

### 3.3 Farm and bird level prevalence of AIV (M-gene) H5 and H9 subtypes in turkey farms

**Figure 5:**
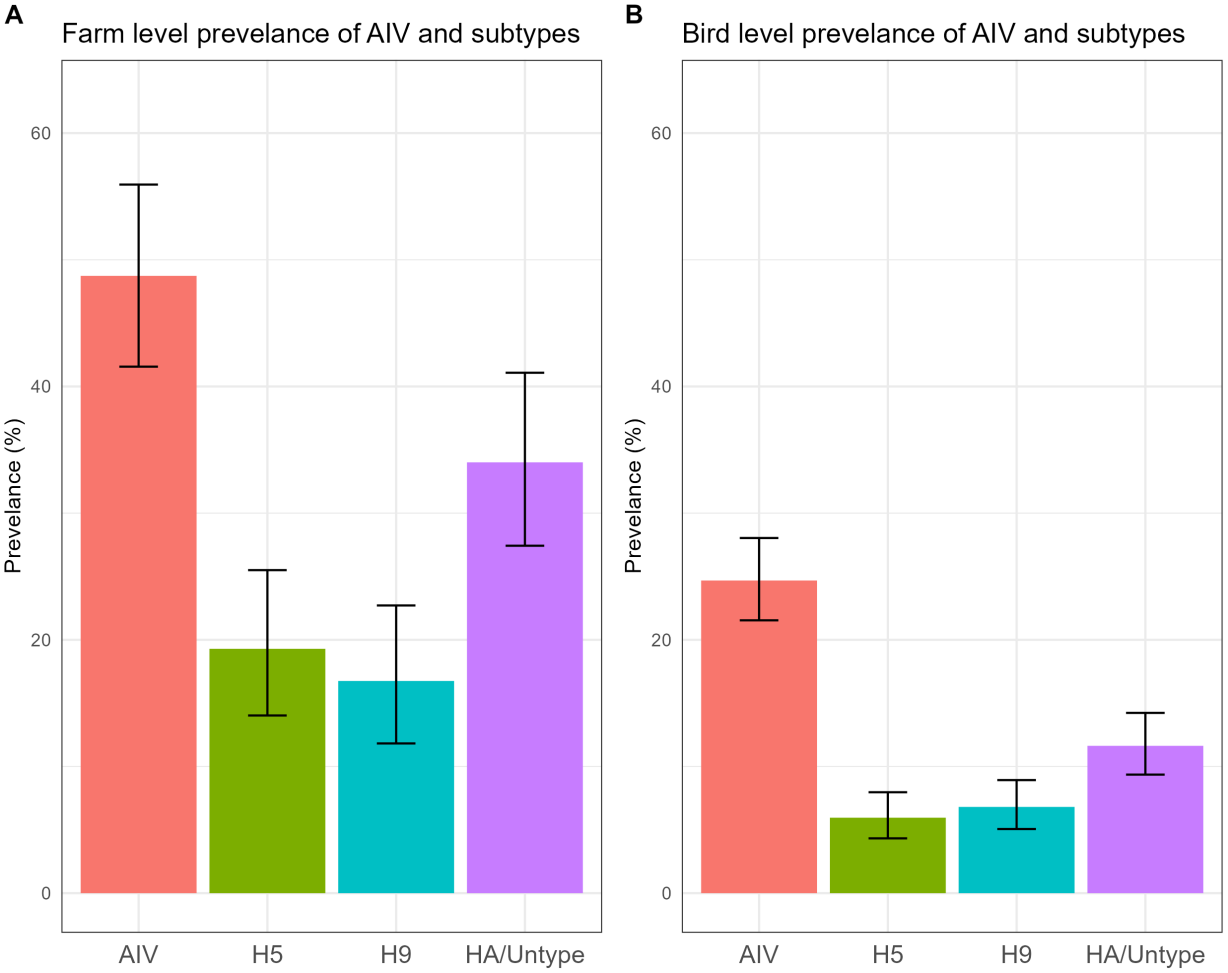
Farm level **(A)** and bird level **(B)** prevalence of AIV and subtypes, along with their 95% CI, is plotted.

The data unveiled an alarmingly high prevalence of AIV and its subtypes within turkey farms. We found that 48.73% (95% CI: 41.56–55.94) (Figure 5A) of the investigated turkey farms were infected with AIV (M gene), indicating that nearly half of the sampled turkey farms had at least one AIV-positive poultry. Additionally, 19.29% (95% CI: 14.03–25.50) of farms were found to be positive for the H5 subtype, while 16.75% (95% CI: 11.82–22.71) tested positive for H9. None of the samples were positive for A/H7 virus. Turkey farms showed higher positivity (66.7%) in March compared to other months sampled (Supplementary Figure S1).

A total of 705 swab samples were collected during the study period. The AIV prevalence for individual turkeys was 24.68% (95% CI: 21.54–28.04) (Figure 5B). The probability reveals that one of the four turkeys tested positive for AIV. The bird-level prevalence of H5 and H9 subtypes was 5.95% (95% CI: 4.33–7.97) and 6.81% (95% CI: 5.06–8.93), respectively.

### 3.4 Association of biosecurity practices and AIV infection in turkey farms

**Figure 6:**
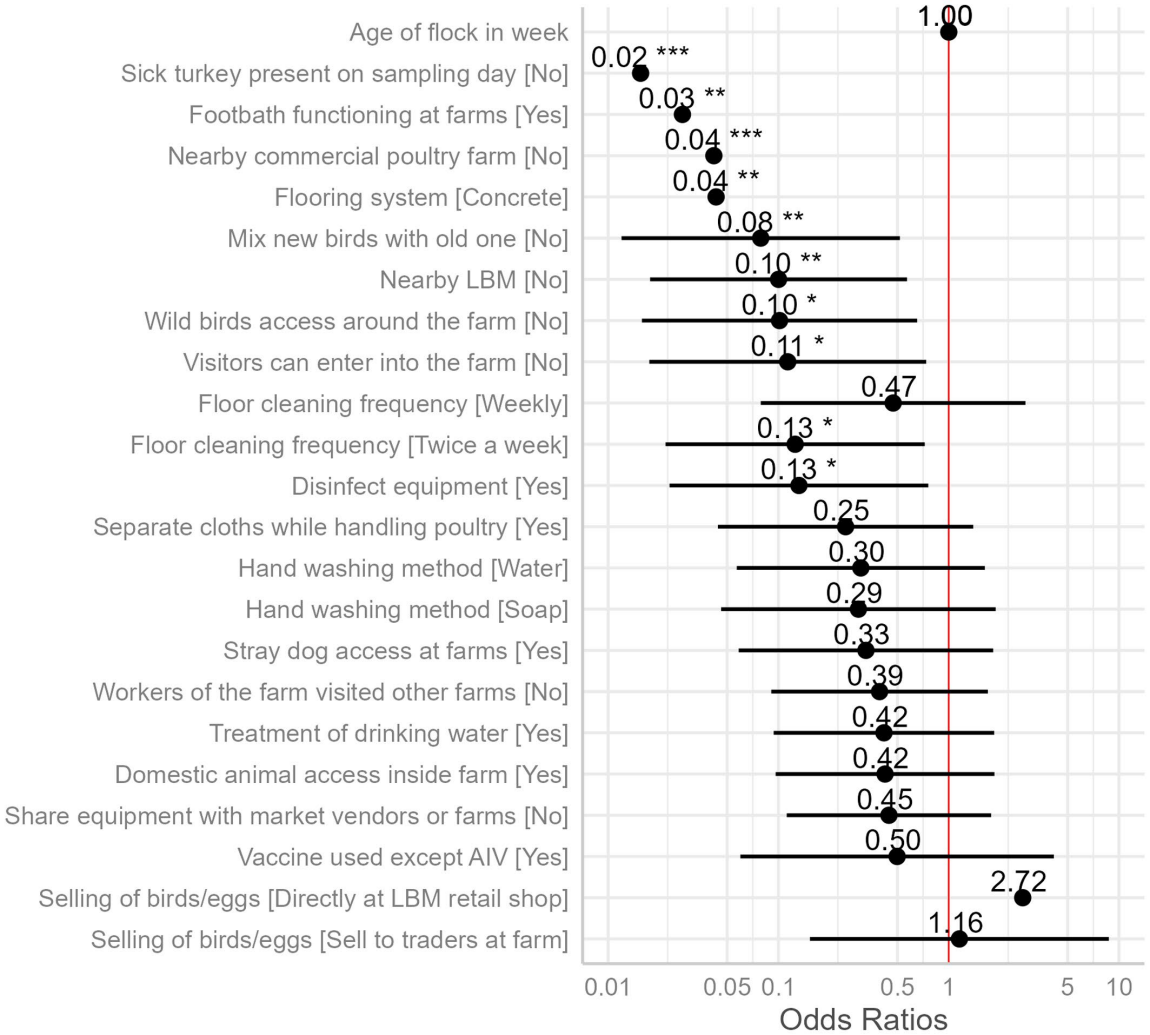
Odds ratios of the presence of AIV as compared to the reference category of each independent variable (intercept, reference category not shown) with 95% confidence intervals and significance stars (*) from mixed effect logistic regression model is plotted. The “neutral” dotted line, i.e., the vertical intercept, indicates no effect (x-axis position 1 for odds ratio).

We identified 10 variables that are significant risk factors for AIV (Table 1). The presence of sick poultry on the farms had the highest odds, with the absence of sick turkeys on the sampling day reducing the odds of AIV by 98% (Figure 6).

**Table 1:** Estimates with standard error and *p*-value of generalized linear mixed effect model to assess the effect of biosecurity practices on the presence/absence of AIV in turkey farms in Bangladesh.

Factors	Category	Estimate	Std. error	Statistic	*p*-value
Age of flock in week		0.001	0.02	0.05	0.96
Sick turkey present on sampling day	Yes	Reference			
	No	−4.17	1.06	−3.92	<0.01
Footbath functioning at farms	No	Reference			
	Yes	−3.6	1.11	−3.26	<0.01
Nearby commercial poultry farm (<500 m)	Yes	Reference			
	No	−3.18	0.93	−3.43	<0.01
Flooring system	Mud	Reference			
	Concrete	−3.15	0.98	−3.2	<0.01
Mix new birds with old one	Yes	Reference			
	No	−2.54	0.96	−2.65	0.01
Nearby LBM (<500 m)	Yes	Reference			
	No	−2.3	0.89	−2.6	0.01
Wild birds access around the farm	Yes	Reference			
	No	−2.29	0.95	−2.41	0.02
Visitors can enter into the farm	Yes	Reference			
	No	−2.18	0.96	−2.28	0.02
Floor cleaning frequency	Twice a month	Reference			
	Weekly	−0.75	0.91	−0.82	0.41
	Twice a week	−2.08	0.89	−2.32	0.02
Disinfect equipment	No	Reference			
	Yes	−2.03	0.89	−2.27	0.02
Separate clothes while handling poultry	No	Reference			
	Yes	−1.39	0.88	−1.58	0.11
Hand washing method	None	Reference			
	Water	−1.19	0.86	−1.39	0.17
	Soap	−1.22	0.95	−1.29	0.2
Stray dog access at farms	No	Reference			
	Yes	−1.12	0.88	−1.27	0.2
Workers of the farm visited other farms	Yes	Reference			
	No	−0.93	0.75	−1.25	0.21
Treatment of drinking water	No	Reference			
	Yes	−0.88	0.76	−1.15	0.25
Domestic animal access inside farm	No	Reference			
	Yes	−0.86	0.75	−1.14	0.25
Share equipment with market vendors or farms	Yes	Reference			
	No	−0.81	0.71	−1.15	0.25
Vaccine used except AIV	No	Reference			
	Yes	−0.7	1.08	−0.64	0.52
Selling of birds/eggs	Both (Traders and LBM)	Reference			
	Directly at LBM retail shop	1	0.9	1.11	0.27
	Sell to traders at farm	0.15	1.03	0.14	0.89

**Figure 7:**
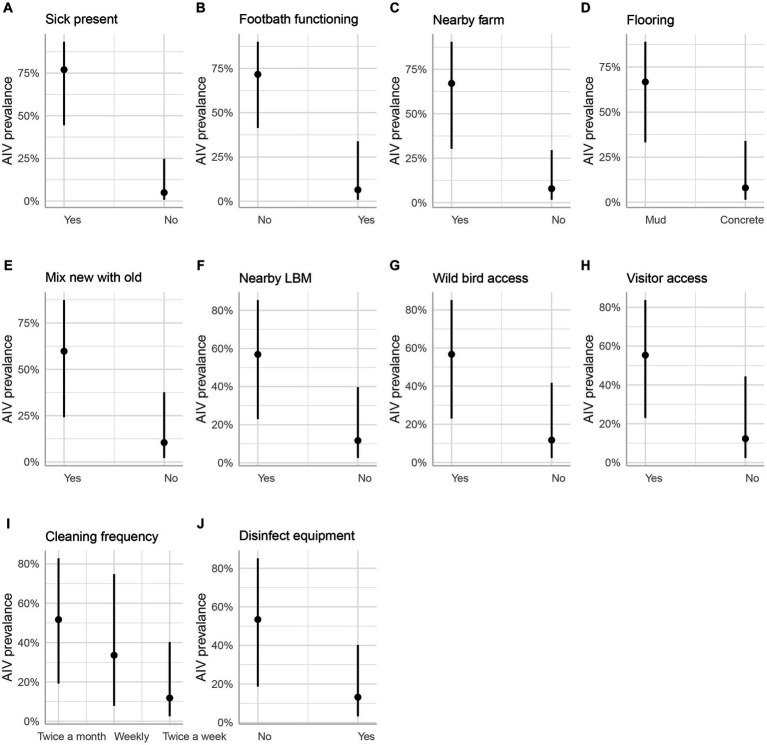
Estimated marginal means (predicted values adjusted for all other effects) and their 95% confidence intervals for 10 significant independent variables (**A-J**, mentioned in the part labels) as a function of the respective explanatory variable. The original data corrected for all other effects are plotted for reference.

In the marginal means plot, the predicted chance of AIV was 77% when sick turkeys were present but only 5% when no turkeys were sick (Figure 7A). The functionality of the footbath at the farm entrance emerged as a significant factor; having a footbath reduced the predicted probability of AIV from 72 to 6% (Figure 7B). Furthermore, the proximity of commercial poultry farms within a 500-meter radius significantly influenced AIV prevalence, with an estimated value of 67%, sharply dropping to 8% in their absence (Figure 7C). Having a concrete floor instead of a muddy floor cut the odds of AIV by 96%. The estimated AIV prevalence on marginal mean plots was 67% on a muddy floor and just 8% on a concrete floor (Figure 7D). Another significant variable with an odds ratio of less than 0.1 was the mixing of newly purchased and previously owned birds Figure 7E). The absence of LBM within a 500-meter radius around the studied turkey farms resulted in a substantial reduction in AIV odds by 90% (Figure 7F).

Wild bird access and visitor access were both identified as possible risks, with odds ratios of 0.10 and 0.11, respectively. If wild birds had access to the farm premises, the prevalence of AIV was estimated to be 57%, but it was just 12% if they did not (Figure 7G). The predicted prevalence rises to 55% if visitors can enter the farm, compared to 12% if they cannot (Figure 7H). The next two factors did not show much difference in categories while predicting the AIV probabilities. For the frequency of floor cleaning, the category of twice-a-week predicted a prevalence of 12%, while the weekly frequency category boosted this prediction to 34%, and the twice-a-month category resulted in a higher prevalence of 52% (Figure 7I). When the farmers used disinfection to clean their equipment (feeding trays and pots), this practice minimized the odds of AIV transmission among poultry by 87%. The use of disinfectants predicted the marginal mean by 53%, whereas the lack of their use predicted a decline in the prevalence by 13% (Figure 7J). Vaccination against other diseases (ND and IBD) except AIV did not show any significant differences in AIV transmission (Figure 6).

## 4 Discussion

We comprehensively evaluated the influence of biosecurity practices on the occurrence of AIV at turkey farms in Bangladesh. A significant percentage of farms were found to be non-compliant with DLS’s biosecurity guidelines. The study also revealed an alarming prevalence of H5 and H9 viruses in turkey farms. However, all of the farms were negative for the H7 subtype. The investigation unveiled 10 risk factors that were significantly associated with the AIV infection in turkey farms. The majority of the farmers in our study were male, between the ages of 30 and 40 years, and held a higher secondary level of education. Because of the recent introduction of turkey farming in Bangladesh, a significant number of young, educated male farmers demonstrated a willingness to undertake the associated risks in this farming. These demographic characteristics match with a previous study conducted in 2017 on turkey farming in Bangladesh (52). Upon comparing the biosecurity guidelines adopted by the Department of Livestock (DLS), Bangladesh (44), we observed that the majority of the turkey farms showed poor hygienic and inadequate biosecurity practices in study areas, which contributed to the spread of the virus and worsened the health status of turkey birds, thereby raising the risk of spillover to humans (53).

Our research findings revealed that an alarming proportion of the farms were infected with AIV, with one in every four turkeys testing positive. Even H5 subtypes had a one-fifth probability, and H9 had a one-sixth probability of being positive. This may be due to the fact that turkeys are highly susceptible to AIV infection, and inadequate biosecurity measures contribute to the dire situation of turkey farms in Bangladesh. The vaccination campaign against AIV in Bangladesh focuses mostly on commercial chicken farms (54), as domestic turkeys have shown significant susceptibility to H5N1 infection. Thus, it is crucial to expand the vaccine effort to include turkey farms to effectively control AIV in Bangladesh.

Biosecurity in poultry farms is the first line of defense for preventing the entry and spread of infectious pathogens, which may greatly impact poultry health, food safety, and economic stability (55). In our study, the emergence and spread of AIV in turkey farms might be attributed to inadequate biosecurity and hygienic measures in the farms. In our study, we have identified 10 farm biosecurity practices that significantly influence AIV transmission in turkey farms, which are as follows: the absence of sick birds, having no nearby poultry farms, using a footbath at the farm entrance, managing farm flooring with concrete, preventing the mixing of new and old birds in the same shed, maintaining a standard distance from LBMs near the farm, avoiding access by wild birds, limiting visitors into the farms, cleaning the farm floor twice a week, and disinfecting farm equipment significantly reduced the risk of AIV infection in turkey farms. Our study revealed that 60% of turkey farms did not have any sick birds on the sample day, which significantly reduced the prevalence of AIV prevalence, and the odds were calculated as small as 0.02 (Figure 6). Sick birds enduring immunological stress can impact the spread of AIV and transmission of the viruses to other healthy birds (56, 57). In Bangladesh, both sick and healthy poultry are kept together in a poultry-rearing system (58), where healthy birds get infections from the sick birds through direct and indirect contact with fomites, droplets, and contaminated water and feed (59), resulting in the higher rate of AIV in sick birds than the healthy birds (14, 57, 60). No sick poultry present on the sampling day is correlated with the mixed farming system (rearing more than one poultry) (Cramer’s V = 0.32), so this variable should be mentioned.

The presence of footbaths in turkey farms plays a crucial role in preventing the spread of AIV by reducing mechanical transmission and spreading of infections inside the farm (61). The introduction of AIV into poultry farms can occur due to contaminated footwear and insufficient decontamination of footbaths (62), where the establishment of footbaths is not commonly practiced in Bangladesh (63, 64). The presence of the footbath at the poultry entrance of the farm significantly reduced the AIV transmission and reported odds as 0.49 in the study by Chaudhry et al. (65), whereas, in our study, it was calculated as 0.03. Consistent use of footbaths with potent disinfectants reduces the risk of introducing infectious pathogens to poultry farms, enhancing biosecurity measures in these premises (66). The presence of footbath was correlated with the grazing system of turkey (Cramer’s V = 0.40) and the cleaning agent used for floor cleaning (Cramer’s V = 0.36), and these variables might have an independent effect on AIV circulation at the farm level.

The absence of neighboring poultry farms has been identified as a significant protective factor associated with lowering the AIV transmission in the turkey farms in our study, where the presence of nearby poultry farms was reported to be a higher risk of AIV transmission in previous studies (67). The close proximity of the other farms plays a significant role in AIV transmission through inter-farm traffic, including the sharing of farm equipment and work staff (68), and through the wind. In the housing management of turkey farming, flooring with concrete reduces AIV transmission in the turkey farms, which is supported by the study by Gompo et al. (69), where the risk has been reported to be 1.23 times higher in the muddy floor than the cemented floor during the H9 outbreak in commercial farms. Muddy flooring system in commercial turkey farms increases the risk of AIV transmission as farmers remove the litter by scraping the mud with sharp-edged equipment and smearing new mud without applying any disinfectant; they allow it to dry for 2–7 days (70). Soil and mud contain rich microorganisms, including pathogenic viruses and bacteria, offering the risk of infection, such as AIV (71). The concrete floor allows better disinfection (72), which is effective in decreasing AIV transmission; in our study, the reduction of the AIV risk was reported at 96%, with a predicted prevalence of AIV of 8%. In addition, the management of sick birds could have an effect on the circulation of AIV and affect the model since Cramer’s V is 0.31 while calculated with the flooring system. The study also reported that separating new from old birds in the turkey farms significantly reduced the occurrence of AIV, with the odds of AIV as low as 0.1. In a previous study by Mumu et al. (36), a significant percentage of turkey mortality due to HPAI H5N1 outbreak was reported on a turkey farm in Bangladesh where they reared different ages of turkeys in a single shed.

No nearby LBMs present by the farms have been reported as significant protective factors for reducing AIV in the turkey farms, as LBMs are the major hub of AIV infection and transmission in the poultry industry (34, 73). In Bangladesh, poultry farms are generally established close to the LBMs (39), which may be a crucial risk of circulating the AIV in the poultry of the farm. Live bird trading is frequent in Bangladesh, as more than 90% of poultry are marketed through LBMs (74). Mixing of different species of birds (chickens, ducks, geese, pigeons, etc.) from different sources (wild birds, backyards, and commercial farms) in LBMs creates a suitable niche for the persistence and perpetuation of AIV (75, 76). In our study, the predicted prevalence of AIV infection was 12%, where no LBMs were established near (<500 m) the turkey farms, with a reduction of odds at 90%. LBMs typically provide foraging opportunities for wild birds and peri-domestic birds, such as crows, sparrows, and starlings (77, 78), where those wild birds visit the poultry farms close to the LBMs and spread the AIV into healthy birds of the commercial poultry farms. We cannot ignore the importance of a disposal system for dead birds as this variable had a higher value of Cramer’s V (= 0.30) with the presence of LBM nearby.

No access to wild birds in the present study has been identified as a significant protective factor in reducing the risk of AIV in turkey farms, with odds equal to 0.10. Wild birds are the natural reservoir of AIV, and they can contaminate the environment of the farm by drooping in and around the farms, contributing to the transmission of AIV (59, 79). The access of wild birds is commonly found in small-scale commercial poultry farms where they scavenge on feeds and water premises of commercial farms (70, 80), which increases the risk of AIV infection from wild birds as HPAIV has been reported sporadically in wild birds in Bangladesh (34, 81). Having access to wild animals within commercial farms had a 5.7 times higher risk of AIV transmission in commercial poultry (82). Mortality of the wild birds surrounding or near the farm area is reported to be the most potent risk factor for HPAI transmission in commercial farms in Bangladesh and India (83, 84). Controlling the access of wild birds in the turkey farms significantly reduced the risk of AIV transmission and the predicted prevalence was reported to be only 12%.

In the present study, no access of visitors into the poultry farms has been reported as a significant protective factor against AIV transmission, whereas the previous studies illustrated that visitors not allowed from other poultry farms and retail markets significantly reduce the odds of AIV transmission (80, 85). In our study, we observed that the predicted prevalence of AIV transmission was reported to be only 12% where there were no access visitors to the turkey farms, whereas, in the previous study by Subedi et al. (86), this risk was reported to be 2.8 times higher in commercial farms where visitors are allowed to sheds during the H9 outbreak. Visitors such as veterinarians and technicians visit multiple farms in a day for Newcastle disease (ND) vaccinations without thorough decontamination, contributing significantly to AIV transmission between farms (87), as ND and AIV frequently co-infect in poultry farms (88). No access to visitors is correlated with the mixed farming system (rearing more than one poultry) (Cramer’s V = 0.32), so this could have an effect on the AIV transmission.

Disinfectant practices in turkey farms have been reported as a significant factor in lowering the predicted AIV infection rate to 40%, with odds equal to 0.13. Poultry farmers use locally available materials such as lime, bleaching powder, and potash as disinfectants, whereas commercial farms practice spraying both inside and outside of the shed, although disinfecting utensils, feed sacks, and equipment used for collecting litter have rarely been practiced (70). Disinfection with those chemical agents is an important element of the biosecurity program against AIV and reduces the chance of infection (89). Cleaning the floor twice a week has been identified as a protective factor against AIV transmission (odds = 0.13) in our study, as it has reported the prediction of an AIV infection rate of only 12%, whereas cleaning twice a month predicted the probability of 52%. Cleaning the floor frequently notably reduced the AIV risk in the previous study (42). Conversely, infrequent cleaning can increase the risk of AIV transmission (81).

The current research has several limitations which need to be taken into account. The results may have been impacted by self-reported data due to biases, such as methodological, social desirability, and memory recall biases. The possibility of recall bias may have affected the replies of farmers. However, we addressed this bias by specifically considering the present chicken production cycle. Furthermore, interviews were conducted with individuals who held major farm ownership positions and were actively engaged in poultry management. As such, these individuals were anticipated to possess comprehensive knowledge regarding the biosecurity practices that were implemented on the farms. In our study, we could not continue the study over the year to take advantage of the seasonal pattern due to time and budget constraints. To address these constraints, future research should utilize longitudinal designs that incorporate a more diverse and representative sample. Additionally, it is important to investigate the previous health concerns and infections that have been experienced in chicken farms.

## 5 Conclusion

Our research uncovered a high prevalence of H5 and H9 subtypes circulating in turkey farms. Furthermore, we have identified certain potential risk factors for AIV transmission that are associated with the failure to adhere to recommended standard hygiene and biosecurity practices on farms. Additionally, we observed that ignoring effective biosecurity measures can exacerbate AIV infection in the farms, whereas implementing them can mitigate the risk. A small amount of additional effort needs to be made to ensure that all farmers employ biosecurity practices since some of them already do so. It is recommended that governments at all levels undertake the task of educating farmers about the benefits associated with investing in biosecurity compliance. Additionally, it is important to highlight the possible hazards linked to non-compliance, particularly regarding the constantly evolving AIV and the risk of spillover to humans. Moreover, it is imperative for governments to provide farmers with comprehensive training in practical and economically feasible biosecurity measures. Our research findings offer pertinent guidance in this regard.

## Data availability statement

The original contributions presented in the study are included in the article/Supplementary material, further inquiries can be directed to the corresponding author.

## Ethics statement

The studies involving humans were approved by Ethics Committee at the Chattogram Veterinary and Animal Sciences University (Protocol: CVASU/Dir(R&E) EC/2019/126(1)). The studies were conducted in accordance with the local legislation and institutional requirements. The participants provided their written informed consent to participate in this study. The animal studies were approved by Animal Experimentation Ethics Committee at Chattogram Veterinary and Animal Sciences University (Protocol: CVASU/Dir(R&E) EC/2019/126(1)). The studies were conducted in accordance with the local legislation and institutional requirements. Written informed consent was obtained from the owners for the participation of their animals in this study.

## Author contributions

AI: Conceptualization, Data curation, Formal analysis, Investigation, Methodology, Software, Visualization, Writing – original draft, Writing – review & editing. MI: Data curation, Formal analysis, Software, Validation, Visualization, Writing – review & editing. PD: Data curation, Methodology, Validation, Writing – review & editing. MAR: Data curation, Investigation, Methodology, Writing – review & editing. AM: Data curation, Investigation, Methodology, Validation, Writing – review & editing. AKMDK: Data curation, Validation, Writing – review & editing. MAS: Investigation, Methodology, Validation, Writing – review & editing. MMH: Data curation, Funding acquisition, Investigation, Project administration, Supervision, Writing – review & editing. MZR: Funding acquisition, Investigation, Methodology, Resources, Software, Writing – review & editing. TS: Funding acquisition, Project administration, Resources, Supervision, Writing – review & editing.

## Supplementary material

Supplementary Figure S1 Distribution of AIV positivity over month in the turkey farms in Bangladesh in 2019. The total number of sampled farms along with months is shown in the X axis, and the Y axis represents the proportion of AIV-positive farms in that given month.
