# Supplementary figures and images for: Association of biosecurity and hygiene practices with avian influenza A/H5 and A/H9 virus infections in turkey farms
Source: Front Vet Sci. 2024 Mar 14;11:1319618. doi: 10.3389/fvets.2024.1319618 (PMC10976562; doi:10.3389/fvets.2024.1319618)

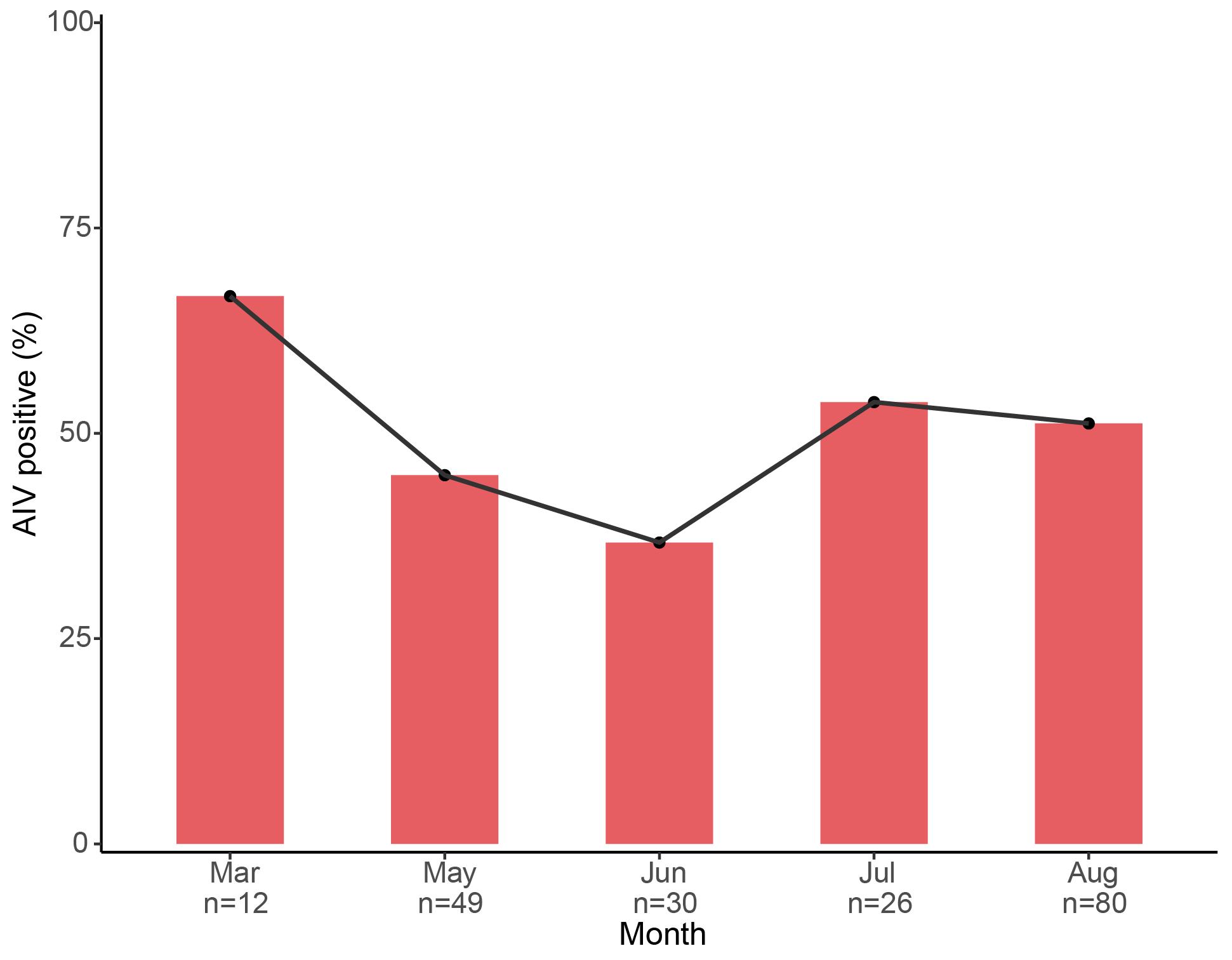

Supplement: Supplementary Figure S1 — Distribution of AIV positivity over month in the turkey farms in Bangladesh in 2019. The total number of sampled farms along with months is shown in the X axis, and the Y axis represents the proportion of AIV-positive farms in that given month. [file Image_1.TIFF]
